# Diabetes Care Knowledge-Practice Gap Among Primary Healthcare Nurses in Saudi Arabia: A Cross-Sectional Assessment of Competency and Training Needs

**DOI:** 10.7759/cureus.116567

**Published:** 2026-09-20

**Authors:** Ayat Ali Al Ali, Abdulrahman Fouad Alkhateeb, Sanaa Sadiq Alsaad, Abdullah Albahussain, Ahlam Saeed Almadhi

**Affiliations:** 1 Family Medicine, Khobar Health Network, Eastern Cluster, Ministry of Health, Al Khobar, SAU; 2 Nursing, Khobar Health Network, Eastern Cluster, Ministry of Health, Al Khobar, SAU

**Keywords:** attitudes, diabetes mellitus, health knowledge, nursing education, practice, primary care nursing, self-efficacy

## Abstract

Introduction: Primary healthcare nurses deliver most routine diabetes contact, yet regional studies rarely test whether nurses self-rated confidence matches objective knowledge. This study assessed diabetes knowledge, self-efficacy, and satisfaction among primary care nurses, and examined concordance between knowledge and self-rated confidence.

Methods: This cross-sectional study was conducted among primary care nurses at 11 primary health centers in Eastern Province, Saudi Arabia. A structured questionnaire combining a 36-item diabetes knowledge test and 17-item self-efficacy scale (5-point Likert) was piloted on 10 respondents (Cronbach's alpha=0.948) before administration to the main analytic sample of 100 nurses. Knowledge and self-efficacy scores were compared using Spearman correlation, Mann-Whitney U, and Kruskal-Wallis tests (p<0.05).

Results: Mean knowledge score was 53.7% (SD 18.9); 59% had poor knowledge (<60% correct). Self-efficacy was moderate-to-high overall (mean 3.66/5). Knowledge and self-efficacy scores were only weakly-to-moderately correlated (Spearman's rho=0.38, p<0.001); 33% of nurses showed knowledge-confidence discordance (20% underconfident, 13% overconfident). Prior diabetes-specific training was associated with significantly higher self-efficacy (p=0.005) but not objective knowledge (p=0.47). Qualification level was associated with knowledge scores on univariate analysis (p=0.016) but not after adjusting for training, experience, and chronic-unit exposure (p=0.088). Glucagon injection technique was the lowest-rated competency (mean 2.57/5); satisfaction with training received was low (mean 2.24/5) and uncorrelated with objective knowledge (p=0.48).

Conclusion: Primary care nurses show a measurable disconnect between diabetes-related confidence and objective knowledge: existing training is associated with strengthened self-assurance without a proportional association with improved knowledge. Structured, competency-verified training targeting glucagon administration and Ramadan-specific counseling is needed to close this gap and safeguard patient outcomes.

## Introduction

Diabetes mellitus is one of the fastest-growing chronic disease burdens worldwide. The International Diabetes Federation’s 11th edition Diabetes Atlas estimated that 589 million adults aged 20-79 years were living with diabetes in 2024, a figure projected to rise substantially by 2050 as population ageing and urbanization accelerate disease incidence across low- and middle-income regions [1]. The Middle East and North Africa region carries a disproportionate share of this burden, and Saudi Arabia in particular has consistently ranked among the countries with the highest national prevalence of type 2 diabetes mellitus (T2DM), with pooled estimates from systematic reviews placing prevalence in the range of 16-28% of the adult population over the past two decades [2]. This scale of disease places sustained, day-to-day management demands on the primary healthcare workforce, and nurses - not physicians or specialist diabetes educators - form the frontline of that workforce, providing glucose monitoring, insulin administration, hypoglycemia response, and the bulk of patient education encounters [3].

Diabetes self-management education and support (DSMES), delivered predominantly by nursing staff, is recognized by international consensus as a foundational, evidence-based component of diabetes care that improves glycemic control and reduces complication risk [3]. Because nurses are expected to deliver this education, professional bodies increasingly call for defined “core competencies” for diabetes-care nursing roles [4]. However, the assumption that nurses possess adequate diabetes knowledge to fulfil this educator role has repeatedly been challenged: studies in pediatric and school-nursing settings have documented a measurable gap between what nurses believe they know about diabetes and what they can demonstrate on objective knowledge testing [5], and reviews of non-endocrinology nursing staff report persistently low or inconsistent diabetes knowledge scores alongside unmet training needs [6]. A recent systematic review and meta-analysis of nursing-student diabetes education similarly concluded that structured, competency-based curricula are inconsistently applied and evaluated across training programmes [7].

Within Saudi Arabia specifically, evidence on nurses’ diabetes-care readiness remains limited and points in a concerning direction. A cross-sectional study of nurses in Hail province found a marked disparity between nurses’ perceived and their actual (tested) diabetes knowledge, with actual knowledge substantially lower than nurses’ self-rated confidence would predict [8]. Comparable findings from primary healthcare nurses in other health systems show that self-efficacy in diabetes self-management support does not necessarily track with objective knowledge, and that these two constructs behave as partially independent dimensions of clinical readiness rather than a single underlying competency [9]. Confidence itself has been shown to depend heavily on prior device-specific or task-specific training rather than on generalized diabetes knowledge [10], and hospital-based studies of healthcare professionals managing T2DM have likewise reported that self-rated competence and confidence do not reliably correspond to guideline-concordant clinical performance [11].

Despite this accumulating international evidence that knowledge and confidence can diverge, no published study in Saudi Arabia - or, to our knowledge, in the wider Gulf region - has simultaneously measured primary care nurses’ objective diabetes knowledge and their self-rated competence across a matched set of clinical domains within a single cohort, enabling direct assessment of knowledge-confidence concordance rather than either construct in isolation. This represents a strong and clinically consequential literature gap: if nurses who feel confident in diabetes care are not, in fact, objectively competent, the resulting overconfidence carries direct patient-safety implications for tasks nurses perform independently, such as insulin dosing, glucagon administration in hypoglycemic emergencies, and hypoglycemia recognition. Furthermore, no prior Saudi study has evaluated nurses’ self-rated preparedness for diabetes counseling during Ramadan fasting, despite fasting-related glycemic complications being a well-recognized, regionally distinct clinical priority that is largely absent from international nursing-knowledge instruments.

This study addresses that gap with high novelty on three fronts. First, it is the first study in Saudi Arabia to simultaneously quantify primary care nurses’ objective diabetes knowledge and their self-efficacy across matched clinical domains, allowing direct classification of nurses into concordant, overconfident, and underconfident subgroups rather than reporting either construct alone. Second, it incorporates a Ramadan-specific diabetes counseling competency item, addressing a regionally distinct clinical demand that existing international nursing-knowledge instruments do not capture. Third, by linking knowledge and confidence outcomes to training history, years of chronic-disease-specific experience, and professional qualification level, the study identifies which structural and educational factors predict competency gaps, yielding actionable targets for workforce training rather than a purely descriptive prevalence estimate.

The primary objective of this study was to quantify baseline objective diabetes knowledge and self-efficacy scores among primary care nurses regarding diabetes-related care. Secondary, exploratory objectives were to evaluate the concordance between objective knowledge and self-rated self-efficacy using a median-split taxonomy, and to examine demographic and training-related predictors of these outcomes. The specific objectives were: (1) to measure primary care nurses’ objective knowledge of diabetes pathophysiology, glycemic targets, insulin therapy, hypoglycemia and hyperglycemia management, and diabetic foot care using a structured knowledge instrument; (2) to evaluate nurses’ self-efficacy across core diabetes-care tasks, including glucose monitoring, insulin and glucagon administration, patient education, and Ramadan-specific glycemic counseling; (3) to determine the degree of concordance versus discordance between objective knowledge scores and self-rated confidence, identifying the prevalence of overconfidence and under confidence; (4) to identify demographic, educational, and training-related predictors - including years of experience, prior diabetes-specific training, and professional qualification - associated with knowledge and confidence outcomes; and (5) to assess nurses’ satisfaction with their current knowledge, practice, and training in diabetes care.

## Materials and methods

Study design

This was an analytical cross-sectional study conducted among nurses working in primary healthcare settings within Khobar Health Network, Eastern Cluster, Ministry of Health, Saudi Arabia. A cross-sectional design was chosen as appropriate for a first exploratory assessment of knowledge-confidence concordance in this population, given the absence of prior comparable data and the descriptive, hypothesis-generating aim of the study. Data were collected using a self-administered, structured electronic questionnaire. This study is reported in accordance with the Strengthening the Reporting of Observational Studies in Epidemiology (STROBE) guidelines for cross-sectional studies.

Study population and sampling

Data were collected from May 17, 2025 to August 26, 2025 across all 11 primary health centers and the affiliated urgent care center of Khobar Health Network. Eligible participants were all registered nurses, nursing specialists, nursing technicians, and nursing assistants working in primary health care centers of Khobar Health Network, irrespective of whether they were currently or had previously been assigned to a chronic-disease (diabetes) care activity. Nurses working in specialty clinics within Khobar Health Network, and nurses working in other health networks, were excluded. A convenience sample was recruited by visiting all study sites; the target sample size (n=110) was calculated from the eligible population of 153 nurses using a 95% confidence level and 5% margin of error to ensure adequate precision, though probability sampling was not employed. Of 153 eligible nurses, 110 completed the questionnaire (response rate 71.9%); data on non-responders' characteristics were not available for comparison. The first 10 respondents were designated a priori as a reliability pilot subsample for internal-consistency testing of the self-efficacy scale (see Reliability Testing, below); this pilot size followed conventional practice for preliminary Cronbach's-alpha estimation (commonly 10-30 respondents) and, despite its modest size, yielded an alpha coefficient (0.948) closely consistent with that of the full analytic sample (0.961), supporting its adequacy for this purpose. These 10 respondents were excluded from the main analysis. The remaining 100 respondents constituted the main analytic sample for all descriptive, correlational, and comparative analyses reported here. There were no missing responses among the 100 nurses in the main analytic sample.

Instrument development and adaptation

The questionnaire comprised four sections: (a) sociodemographic and professional characteristics (age, gender, years of nursing experience, professional qualification, chronic-disease-unit work history, and prior diabetes-specific training); (b) a 36-item multiple-choice diabetes knowledge test covering diabetes pathophysiology and classification, diagnostic and glycemic targets, insulin therapy and injection technique, hypoglycemia and hyperglycemia recognition and management, diabetic ketoacidosis, long-term complications, diabetic foot care, exercise and nutrition counseling, and glycated hemoglobin (HbA1c) targets, scored as the number of correct responses (range 0-36); (c) a 17-item self-efficacy scale rated on a 5-point Likert format (1 = not at all confident to 5 = completely confident), covering technical skills (glucose monitoring, insulin injection, glucagon injection, hypoglycemia and hyperglycemia identification and management) and patient-education domains (foot care, diet, exercise, self-monitoring, insulin use, home management of hypoglycemia and hyperglycemia, sick-day and exercise-day glycemic response, insulin storage during travel, and Ramadan-specific glycemic counseling); and (d) three items assessing nurses' satisfaction with their own diabetes-related knowledge, practice, and training received, also rated on a 5-point Likert format (see Appendices 1 and 2). All four sections of the questionnaire - sociodemographic and professional characteristics, the knowledge test, the self-efficacy scale, and the satisfaction items - were developed by the authors of this study. The Diabetes Basic Knowledge Test [12] was used solely as a reference for identifying general knowledge domains relevant to diabetes care nursing (e.g., medication management, blood glucose control, foot care); no individual items were directly adopted, adapted, or paraphrased from it. All 36 knowledge items were independently written by the research team. The instrument was translated into Arabic for administration to participants.

This combined knowledge-plus-self-efficacy structure follows the precedent of prior diabetes-nursing instruments that pair an objective, multiple-choice knowledge component - most commonly adapted from the Michigan Diabetes Knowledge Test, originally developed and validated by the University of Michigan Diabetes Research and Training Center [13], and subsequently used, with local adaptation, among primary healthcare nurses in China [14] and among nurses in Hail province, Saudi Arabia [8] - with a separate self-efficacy or performance instrument, as demonstrated in South African primary care nurses using the Diabetes Basic Knowledge Test alongside the Self-Efficacy and Performance in Self-Management Support instrument [9]. The knowledge item pool and reliability-testing approach used here are also consistent with earlier validated diabetes-knowledge questionnaires used across nursing and physician-trainee populations, including a 21-item instrument with reported test-retest reliability of 0.71 used in a multicenter Pakistani study [15], and the Diabetes Knowledge Questionnaire (DKQ-24), originally developed for Mexican American adults with T2DM [16] and subsequently used to evaluate nurse-led diabetes education, covering comparable domains of nutrition, treatment, monitoring, and complications [17]. Content relevance for the Saudi primary-care context, including the Ramadan-specific item, was based on domains established in regional diabetes-educator competency frameworks [4].

Content validity

Content validity and comprehensiveness of the questionnaire were assessed by an expert panel comprising family medicine consultants, diabetologists, endocrinologists, and experienced chronic-disease-care nurses working in sectors outside the study sites. Panel members were selected on the basis of their clinical specialty (family medicine, diabetology/endocrinology) and practical experience supervising chronic-disease nursing care. Each expert independently rated every item for clarity and importance using a 1-10 scale (1 = strongly unclear/unimportant, 10 = strongly clear/important), and provided free-text comments on content coverage. Formal item-level and scale-level Content Validity Index (I-CVI/S-CVI) statistics were not calculated; instead, items receiving low clarity or importance ratings, or specific expert comments, were revised, reworded, or removed prior to piloting based on panel consensus. This qualitative expert-rating and revision process, rather than a quantitative CVI, constitutes the content-validity evidence for this instrument.

Reliability testing

Internal consistency of the 17-item self-efficacy scale was assessed using Cronbach’s alpha, calculated first on the pre-designated pilot subsample of 10 respondents and subsequently on the full remaining analytic sample. The pilot subsample yielded an internal consistency of α = 0.948; internal consistency in the main analytic sample (n=100) was α = 0.961, indicating high internal consistency of the scale in this population, though Cronbach’s alpha assesses internal consistency only and does not establish construct validity or dimensionality, and supporting its use for the main analysis.

Data analysis

Data were analyzed using Python (pandas and SciPy libraries). Categorical variables are presented as frequencies and percentages; continuous variables are presented as means and standard deviations (SD). Knowledge scores were converted to percentages (score/36 × 100) and categorized as poor (<60%), moderate (60-79%), or good (≥80%) using Bloom’s cut-off point [18]. Self-efficacy item scores were averaged to a per-item mean (range 1-5) and summed to a total score (range 17-85). The association between knowledge and self-efficacy scores was tested using Spearman’s rank correlation coefficient (rho) as the primary measure, given the non-normal distribution of both variables, with Pearson’s correlation coefficient additionally reported for comparability with prior literature in this field. Knowledge-confidence concordance was classified using a median-split approach: respondents scoring at or above the sample median on both knowledge and self-efficacy were classified as “concordant-high”; those below the median on both were “concordant-low”; those below the knowledge median but at or above the self-efficacy median were classified as “overconfident”; and those at or above the knowledge median but below the self-efficacy median were classified as “underconfident.” Group comparisons of knowledge and self-efficacy scores by prior training, chronic-disease-unit exposure, and professional qualification were conducted using the Mann-Whitney U test (two groups) or Kruskal-Wallis H test (three or more groups), as the knowledge-score and self-efficacy distributions did not meet normality assumptions. Following the significant Kruskal-Wallis test for qualification, post-hoc pairwise comparisons were conducted using Dunn’s test with Bonferroni correction. To account for multiple comparisons across the 12 significance tests reported (the four knowledge-score group comparisons and one self-efficacy group comparison, and the six satisfaction-domain correlations plus the primary knowledge-self-efficacy correlation reported, p-values were additionally adjusted using the Benjamini-Hochberg false discovery rate (FDR) procedure; results are considered robust only where they remained significant after this adjustment. To assess whether each predictor's association with knowledge held independently of the others, a multiple linear regression model was subsequently fitted with knowledge percentage as the outcome and training status, qualification (Specialist vs. Technician; the Nursing Assistant subgroup, n=3, was excluded due to insufficient size for a stable estimate), years of experience (group midpoint, used as a pragmatic continuous approximation of the banded experience categories collected), and chronic-disease-unit exposure as simultaneous predictors; model assumptions (linearity, homoscedasticity, residual normality, and multicollinearity via variance inflation factors) were checked and found acceptable. A two-tailed p-value <0.05 (raw or FDR-adjusted, as specified) was considered statistically significant throughout.

Ethical considerations

Ethics approval for this study was obtained from the Institutional Review Board (IRB) of Khobar Governmental Hospital, Eastern Health Cluster, Ministry of Health, Saudi Arabia, under IRB registration number H-05-KH-130 (Protocol No. CRO-1), approved on May 6, 2025, prior to the commencement of data collection. This prospective cross-sectional study utilized a structured, self-administered questionnaire completed anonymously by primary care nurses; no patient data or personally identifying participant information were collected. The requirement for documented informed consent was waived by the IRB because the study involved anonymous survey data and posed minimal risk to participants. All procedures were conducted in accordance with relevant institutional guidelines and regulations and the ethical principles of the Declaration of Helsinki. Study data were stored securely for a minimum of five years following study completion, per institutional policy.

## Results

Sociodemographic and professional characteristics

Of the 100 nurses in the main analytic sample, the majority were female (98%) and aged 31-40 years (55%), with over one-third (35%) holding more than 15 years of nursing experience despite this, only 36% reported having received any prior diabetes-specific training, and slightly over half (54%) had ever worked, currently or previously, in a chronic-disease care activity (Table 1). This pattern - a predominantly experienced but under-trained workforce - sets up the central finding of this study: that years on the job did not substitute for structured diabetes education, a relationship examined formally in the predictors analysis below.

**Table 1:** Sociodemographic and professional characteristics of primary care nurses (N=100) Values are n (%) unless otherwise stated. Percentages for subcategories are calculated on the total sample (N=100).

Variable	n	%
Age group (years)		
21–30	4	4.0
31–40	55	55.0
41–50	31	31.0
51–60	10	10.0
Gender		
Female	98	98.0
Male	2	2.0
Years of nursing experience		
2–5	6	6.0
6–10	13	13.0
11–15	43	43.0
15–20	15	15.0
>20	23	23.0
Professional qualification		
Nursing Specialist	29	29.0
Nursing Technician	65	65.0
Other qualification (Nursing Assistant/unspecified)	6	6.0
Ever worked in a chronic-disease (diabetes) unit	54	54.0
Received prior diabetes-specific training	36	36.0
Personal history of diabetes	8	8.0
First-degree relative with diabetes	45	45.0

Objective diabetes knowledge

Objective diabetes knowledge was, on average, weak across the sample. The mean knowledge score was 19.3 of 36 items correct (53.7%, SD 18.9), with scores ranging from 1 (2.8%) to 35 (97.2%) - a spread wide enough to indicate substantial heterogeneity in preparedness rather than a uniformly moderate workforce. When scores were categorized against conventional competency cut-offs, 59% of nurses fell into the poor-knowledge band (<60% correct), compared with only 6% classified as good (≥80%) (Table 2). This distribution is analytically important: it means that a majority-poor knowledge base coexisted with a self-efficacy profile that, as shown below, was not correspondingly low, foreshadowing the confidence-knowledge discordance formally quantified in the concordance analysis.

**Table 2:** Objective diabetes knowledge scores and categorization (N=100) SD, standard deviation. Knowledge percentage calculated as (score/36) × 100.

Knowledge Measure	Value
Mean score (of 36 items), mean ± SD	19.3 ± 6.8
Mean percentage correct, mean ± SD	53.7% ± 18.9%
Median percentage correct	55.6%
Minimum – Maximum percentage correct	2.8% – 97.2%
Knowledge category: Poor (<60%), n (%)	59 (59.0%)
Knowledge category: Moderate (60–79%), n (%)	35 (35.0%)
Knowledge category: Good (≥80%), n (%)	6 (6.0%)

Self-efficacy across diabetes-care domains

In contrast to the predominantly poor objective knowledge, self-rated competence clustered in the moderate-to-high range: 48% of nurses rated themselves as highly confident (per-item mean ≥4/5) and only 22% as low confidence (<3/5) (Table 3). Domain-level analysis showed no meaningful difference between the technical/clinical skills domain (glucose monitoring, insulin and glucagon injection, hypo-/hyperglycemia identification; mean 3.68/5) and the patient-education domain (mean 3.65/5; Wilcoxon p=0.478), indicating that nurses did not rate themselves systematically more confident in “hands-on” tasks than in counseling tasks. However, item-level inspection revealed a markedly uneven competence profile rather than a flat, uniformly moderate one. Correct glucagon injection technique was the single lowest-rated item in the entire instrument (mean 2.57/5, SD 1.70) - nearly a full scale-point below the next-lowest items, which concerned responding to glycemic changes during sick days and exercise (means 3.17-3.21/5). Ramadan-specific glycemic counseling also ranked among the five lowest-rated competencies (mean 3.35/5), despite its clear regional clinical relevance. The two highest-rated items were basic technical skills - glucose measurement via glucometer (mean 4.46/5) and insulin injection technique (mean 4.30/5) - suggesting that confidence was concentrated in the most routinely practiced tasks and dropped sharply for lower-frequency, higher-stakes interventions such as glucagon administration.

**Table 3:** Self-efficacy: overall categorization and the five lowest- and highest-rated items (N=100) SD, standard deviation. All items scored on a 5-point Likert scale (1 = not at all confident, 5 = completely confident).

Item	Value
Overall per-item mean score (of 5), mean ± SD	3.66 ± 1.30 (item-level)
Total scale score (of 85), mean ± SD	62.2 ± 17.5
Category: High confidence (≥4/5), n (%)	48 (48.0%)
Category: Moderate confidence (3–3.99/5), n (%)	30 (30.0%)
Category: Low confidence (<3/5), n (%)	22 (22.0%)
Clinical/technical-skills domain mean (6 items)	3.68 ± 0.98
Patient-education domain mean (11 items)	3.65 ± 1.12
Lowest-rated item: Correct glucagon injection technique	2.57 ± 1.70
2nd lowest: Responding to glycemic changes during exercise	3.17 ± 1.40
3rd lowest: Responding to glycemic changes during sick days	3.21 ± 1.34
4th lowest: Identifying signs/symptoms of hyperglycemia	3.31 ± 1.28
5th lowest: Ramadan-specific glycemic counseling	3.35 ± 1.32
Highest-rated item: Glucose measurement via glucometer	4.46 ± 1.12
2nd highest: Correct insulin injection technique	4.30 ± 1.22

Concordance between objective knowledge and self-rated confidence

Objective knowledge and self-rated confidence were only weakly to moderately correlated (Spearman’s rho=0.38, p<0.001; Pearson’s r=0.45, p<0.001), indicating that confidence explained well under a quarter of the variance in actual knowledge (r²≈21%). Classifying nurses by a median split of both measures showed that only 67% fell into concordant categories (37% concordant-high, 30% concordant-low); the remaining 33% showed outright discordance between what they knew and how confident they felt (Table 4). Critically, this discordance was not symmetric in a reassuring direction: underconfidence (20%, i.e., adequate knowledge paired with low self-rated confidence) was more common than overconfidence (13%, i.e., poor knowledge paired with high self-rated confidence), but the presence of a meaningful overconfident subgroup - more than one in eight nurses believing themselves competent despite testing below the knowledge median - is the pattern of greatest patient-safety concern, since these are the nurses least likely to seek supervision or additional training precisely where it is most needed.

**Table 4:** Concordance between objective knowledge and self-rated confidence, classified by median split (N=100) Median knowledge score = 55.6% correct; median self-efficacy per-item score = 3.91/5. Spearman’s rho between continuous knowledge percentage and self-efficacy mean = 0.38 (p<0.001).

Concordance Category	Definition	n	%
Concordant-High	Knowledge ≥ median AND self-efficacy ≥ median	37	37.0
Concordant-Low	Knowledge < median AND self-efficacy < median	30	30.0
Underconfident	Knowledge ≥ median but self-efficacy < median	20	20.0
Overconfident	Knowledge < median but self-efficacy ≥ median	13	13.0

Predictors of knowledge and self-efficacy

Group comparisons revealed a pattern of associations consistent with a training-knowledge dissociation rather than uniformly deficient training; the terms “knowledge-practice gap” and “overconfidence”/“underconfidence” used elsewhere in this manuscript refer to self-reported measures and a sample-relative median-split classification, not to directly assessed clinical performance or patient-safety outcomes. Prior diabetes-specific training was associated with significantly higher self-efficacy (mean 4.03 vs. 3.45 among untrained nurses; Mann-Whitney p=0.005, FDR-adjusted p=0.010), a moderate effect (rank-biserial r=−0.34), but showed no significant association with objective knowledge (mean 55.8% vs. 52.5%; p=0.470), with a negligible effect size (r=−0.09) suggesting this is a genuine null result rather than an underpowered near-miss. In other words, training appeared to build confidence without proportionally building the underlying knowledge base it was presumably intended to transmit. Professional qualification level, by contrast, was the one variable significantly associated with objective knowledge (Kruskal-Wallis H=8.30, p=0.016, FDR-adjusted p=0.024; η²=0.067, a small-to-moderate effect explaining roughly 7% of knowledge-score variance): Nursing Specialists scored highest (mean 59.6%), followed by Nursing Technicians (52.0%) and Nursing Assistants (41.7%), suggesting that foundational academic preparation contributed more to actual knowledge than post-qualification training exposure did. Neither years of nursing experience (H=7.24, p=0.124, FDR-adjusted p=0.165; η²=0.034) nor prior chronic-disease-unit work experience (p=0.266, FDR-adjusted p=0.319; r=−0.13) was significantly associated with knowledge scores, indicating that time spent in the role - in the absence of structured, competency-verified education - did not translate into measurably better diabetes knowledge (Table 5).

**Table 5:** Group comparisons of knowledge and self-efficacy scores by training, qualification, experience, and chronic-disease-unit exposure (N=100) Statistically significant at p<0.05. Test statistics: U (Mann-Whitney, 2 groups) or H (Kruskal-Wallis, ≥3 groups). p-values shown as raw, then false discovery rate (FDR)-adjusted (Benjamini-Hochberg). Effect sizes: rank-biserial r (Mann-Whitney) or eta-squared η² (Kruskal-Wallis); both interpreted as negligible/small/moderate/large by conventional thresholds. Self-efficacy comparisons were computed only for prior diabetes training, the predictor of primary interest for this outcome; self-efficacy was not compared across chronic-unit exposure, qualification, or experience subgroups, as these were not part of the primary hypotheses for that measure.

Outcome	Predictor	Group	N	Mean	Test statistic	Effect size	Raw p	FDR-adj p
Knowledge (%)	Prior diabetes training	Yes	36	55.8	U=1253.0	r=−0.09	0.470	0.482
		No	64	52.5				
Knowledge (%)	Chronic-unit exposure	Ever/current	54	55.9	U=1403.0	r=−0.13	0.266	0.319
		Never	46	51.0				
Knowledge (%)	Qualification	Specialist	29	59.6	H=8.30	η²=0.067	0.016*	0.024*
		Technician	65	52.0				
		Assistant	3	41.7				
Knowledge (%)	Years of experience	2-5	6	44.9	H=7.24	η²=0.034	0.124	0.165
		6-10	13	56.4				
		11-15	43	57.4				
		15-20	15	45.2				
		>20	23	52.9				
Self-Efficacy (/5)	Prior diabetes training	Yes	36	4.03	U=1544.5	r=−0.34	0.005*	0.010*
		No	64	3.45				

When these four predictors were entered simultaneously into a multivariable linear regression model, however, the qualification effect did not reach statistical significance (Technician vs. Specialist: β=−8.46, 95% CI −18.23 to 1.30, p=0.088), and the overall model explained little variance in knowledge scores (R²=0.055, adjusted R²=0.012; F=1.29, p=0.279). This suggests that the univariate qualification effect may be partly confounded by its correlation with the other predictors, and that no single measured factor robustly and independently explained the observed variation in knowledge - a finding that itself is informative, since it indicates the knowledge gap is not attributable to any one easily targeted structural variable and likely reflects broader curricular or training-delivery factors not captured by these demographic and experience measures (Table 6).

**Table 6:** Multivariable linear regression predicting objective knowledge percentage (n=94) Model R²=0.055 (adjusted R²=0.012); overall model not statistically significant, F(4,89)=1.29, p=0.279. The regression sample (n=94) excludes the Nursing Assistant subgroup (n=3) due to insufficient size for a stable estimate, together with an additional three respondents whose professional qualification did not correspond to a mapped category (two “Other/unspecified” and one Midwifery Technician).

Predictor	β (unstandardized)	95% CI	p-value
Intercept	55.78	44.15 to 67.42	<0.001
Qualification: Technician (ref: Specialist)	-8.46	-18.23 to 1.30	0.088
Prior diabetes training (yes vs. no)	3.15	-5.44 to 11.74	0.468
Years of experience (per year)	0.09	-0.75 to 0.92	0.838
Chronic-unit exposure (ever/current vs. never)	3.59	-4.75 to 11.93	0.394

Satisfaction with knowledge, practice, and training

Nurses’ self-rated satisfaction mirrored the confidence-knowledge dissociation observed above. Satisfaction with one’s own diabetes knowledge (mean 3.26/5) and practice (mean 3.27/5) were both significantly, though only moderately, correlated with objective knowledge (rho=0.34 and 0.27 respectively, both p<0.01) and were strongly correlated with self-efficacy (rho=0.71 and 0.73 respectively, both p<0.001) - confirming that satisfaction tracks how confident nurses feel rather than what they objectively know. Satisfaction with training received was markedly lower than the other two satisfaction domains (mean 2.24/5) and, notably, showed no significant correlation with objective knowledge whatsoever (rho=0.07, p=0.482), while still correlating moderately with self-efficacy (rho=0.42, p<0.001). This dissociation - low and knowledge-independent training satisfaction, alongside a self-efficacy boost from training exposure - reinforces the Table 5 finding that existing training is associated with higher confidence in a manner that is measurably decoupled from objective knowledge gains (Table 7).

**Table 7:** Satisfaction with diabetes-related knowledge, practice, and training, and correlation with objective knowledge and self-efficacy (N=100) Statistically significant at p<0.05. rho, Spearman's rank correlation coefficient; SD, standard deviation. Values in parentheses are raw p, followed by the Benjamini-Hochberg false discovery rate (FDR)-adjusted p.

Satisfaction Domain	Mean ± SD (of 5)	rho vs. Knowledge (p)	rho vs. Self-Efficacy (p)
Satisfaction with own diabetes knowledge	3.26 ± 1.03	0.34 (0.0005*, 0.0012*)	0.71 (<0.001*, <0.001*)
Satisfaction with own diabetes-care practice	3.27 ± 0.99	0.27 (0.007*, 0.011*)	0.73 (<0.001*, <0.001*)
Satisfaction with diabetes training received	2.24 ± 1.34	0.07 (0.482, 0.482)	0.42 (<0.001*, <0.001*)

## Discussion

Principal findings

This study provides the first direct, within-cohort comparison of objective diabetes knowledge and self-rated confidence among primary care nurses in Saudi Arabia. Three findings stand out analytically rather than merely descriptively. First, a majority-poor objective knowledge base (59% of nurses) coexisted with a moderate-to-high self-efficacy profile, producing measurable discordance in one-third of the sample. Second, prior training was significantly associated with self-efficacy but not with knowledge - a dissociation that suggests, as a hypothesis rather than a demonstrated mechanism, that training in this population may operate through affective or motivational pathways rather than strictly cognitive ones; this interpretation was not directly tested and requires confirmation in future research. Third, foundational qualification level showed a univariate association with knowledge that did not persist after multivariable adjustment, which is consistent with, though does not establish, the possibility that the knowledge deficit is not simply a function of insufficient time-on-task but of how initial professional preparation is structured.

The knowledge-confidence gap in context

The magnitude of discordance observed here (33% of nurses knowledge-confidence mismatched) is consistent with, and extends, earlier work documenting that nurses’ perceived diabetes knowledge frequently diverges from their tested, actual knowledge [19]. What the present concordance analysis adds is directionality: rather than treating “knowledge-practice gap” as a single undifferentiated phenomenon, splitting the sample into over- and under-confident subgroups shows that overconfidence, while less prevalent than underconfidence, is clinically the more hazardous pattern, since overconfident staff are structurally less likely to self-identify a training need [20]. This asymmetry echoes findings from an integrative review of diabetes knowledge among nurses across multiple countries, which concluded that self-assessment alone is an unreliable proxy for competency verification and that knowledge deficits often persist undetected specifically because confidence remains high [21]. Our data reinforce that conclusion with a quantified, sample-specific discordance rate rather than a qualitative impression, which is a methodological contribution this analysis makes beyond prior descriptive reports.

Why training raised confidence but not knowledge

The dissociation between training exposure and knowledge outcomes observed in Table 5 is not, on its face, an intuitive result, and it merits closer analytical scrutiny rather than being read simply as “training failed.” One plausible explanation lies in training format: earlier work on practice nurses’ readiness to deliver diabetes self-management education found that short, non-standardized, or lecture-based training sessions - the format most commonly reported by nurses in comparable Saudi and regional workforces - tend to improve nurses’ comfort and willingness to engage with diabetes education without necessarily producing durable, testable knowledge gains [22]. By contrast, structured, competency-verified programmes explicitly designed around simulation, case review, and expert-led coaching have been shown to raise both confidence and demonstrable clinical performance simultaneously [23], which is precisely the training architecture our findings suggest is currently absent for this cohort. This distinction matters practically: it reframes the policy implication from “provide more training” to “redesign training delivery so that knowledge, not only comfort, is measurably verified before and after the intervention.”

Qualification level as a univariate, but not independently confirmed, predictor of knowledge

That professional qualification showed the strongest univariate association with objective knowledge, exceeding both years of clinical experience and training exposure, is analytically suggestive, tentatively shifting attribution away from an easily assumed "experience deficit" narrative toward a curricular one. Comparable long-term-care nursing research has similarly found that formal qualification level, more than tenure, differentiates nurses' diabetes-management knowledge, attributing the gap to differences in pre-service curricular depth rather than to on-the-job learning opportunities [24]. If experience alone were sufficient to build diabetes knowledge, the absence of a significant experience gradient in our data (p=0.124) would be difficult to explain; instead, the pattern is consistent with a "plateau" model in which nurses acquire task familiarity early but do not continue to accumulate testable knowledge without deliberate, structured re-education - a phenomenon previously described in evaluations of nurse-led psychological and educational competency programmes, where competency gains required explicit, assessed curricula rather than passive years-in-role accrual [25]. This interpretation, however, warrants caution: the qualification effect did not persist in the multivariable model (p=0.088), and the low variance explained overall (R²=0.055) indicates that qualification level alone is an incomplete account of the knowledge gap. The curricular hypothesis outlined above therefore remains plausible and consistent with the univariate pattern, but should be treated as hypothesis-generating rather than confirmed, pending replication in a larger sample powered to detect independent effects after adjustment.

Glucagon and Ramadan counseling: two high-stakes, low-confidence domains

The identification of correct glucagon injection technique as the single lowest-rated competency, nearly a full point below the next-lowest items, is a novel and clinically urgent finding that has not, to our knowledge, been isolated as a discrete risk domain in prior Saudi nursing surveys. Glucagon administration is precisely the skill most needed during a low-frequency, high-acuity hypoglycemic emergency in an unconscious patient - a scenario where confidence built from routine, frequently practiced tasks such as glucose monitoring or insulin injection offers no protective transfer. Assessments of the economic and clinical value of structured diabetes education programmes have consistently emphasized that skills used rarely in practice decay fastest and require deliberate refresher training to remain reliable [26], a pattern our item-level data reproduce almost exactly. Similarly, the low self-rated confidence for Ramadan-specific glycemic counseling (ranked fifth-lowest overall) is a gap with no precedent quantification in the literature we are aware of, despite broad recognition that comprehensive diabetes education and support are associated with better linkage to ongoing clinical care and more consistent self-management behavior across variable circumstances such as fasting [27]. The clustering of the two lowest-confidence domains around low-frequency, high-consequence, and culturally-specific scenarios - rather than around routine daily tasks - suggests that current training curricula are organized around common-case exposure rather than around risk-weighted clinical priority, a mismatch with direct implications for curriculum redesign.

Clinical and public health implications

These findings may carry relevance beyond this single cohort, though direct extrapolation to the broader Saudi healthcare system should be made cautiously given the single-network, convenience-sampled design. Meta-analytic evidence has repeatedly shown that effective diabetes self-management education delivered by adequately prepared staff reduces all-cause mortality risk among people with T2DM [28], while professional consensus statements emphasize that psychosocial and educational care quality - not merely biomedical management - depends on the competence of the frontline staff delivering it [29]. In health systems structurally similar to Saudi Arabia’s, primary care-based diabetes self-management surveys in Oman have likewise identified nurse-delivered education as a persistently under-resourced link in the regional chronic-care pathway [30], suggesting the pattern identified here may not be isolated to a single facility network, though this remains to be confirmed in multi-network samples. Given that meta-analyses of self-management education consistently link improved glycemic control to the quality, not merely the presence, of educational delivery [31], and that group-based, structured diabetes self-management education programmes outperform routine, unstructured approaches [32], the corrective indicated by our data is not simply “more training hours” but a shift toward structured, competency-verified, simulation-based training modules explicitly targeting glucagon administration and Ramadan-specific counseling, with pre- and post-training knowledge testing built in rather than relying on self-reported comfort as a proxy for readiness. From a health-systems perspective, this also aligns with the broader public-health argument that investment in chronic-disease workforce capacity is among the most cost-effective interventions available to reduce the long-term burden of non-communicable disease [33].

Strengths and limitations

This study’s principal strength is its within-cohort concordance design, which allowed direct classification of over- and under-confidence rather than relying on separately reported knowledge and confidence prevalence figures drawn from different studies or populations. The instrument’s high internal consistency (α = 0.948-0.961) further supports the reliability of the self-efficacy measurements on which the concordance classification depends. Several limitations should nonetheless be acknowledged. The cross-sectional design precludes causal inference regarding the direction of the training-self-efficacy association observed. Participants were recruited via convenience sampling rather than probability sampling, which may introduce selection and nonresponse bias and limits the generalizability of findings. The sample was drawn from a single primary healthcare network and was predominantly female and Nursing-Technician-qualified, which may further limit generalizability to more specialist-heavy or mixed-gender nursing workforces elsewhere in Saudi Arabia. The author-developed questionnaire has limited external validation evidence beyond the content-validity review described in Methods. Self-efficacy was measured using a self-report Likert scale rather than by direct observation of clinical performance, meaning that even the “concordant-high” subgroup’s self-rated confidence has not been externally validated against observed practice, and the “overconfident”/“underconfident” classifications, based on a sample-relative median split, do not by themselves establish true overconfidence or underconfidence in a clinical sense; nor did this study directly assess clinical performance or patient-safety outcomes. The multivariable regression model explained little variance in knowledge scores and did not reach overall statistical significance, limiting confidence in any individual predictor identified through univariate analysis. Finally, the modest overall sample size (N=100) limited statistical power for multivariable modeling of predictors, and the reliability pilot subsample (n=10) is small by conventional psychometric standards, even though the resulting alpha coefficients were consistent with those obtained in the full analytic sample.

## Conclusions

Primary care nurses in this Saudi cohort demonstrated a majority-poor objective knowledge of diabetes care alongside disproportionately higher self-rated confidence, with one in three nurses showing outright knowledge-confidence discordance. Existing diabetes-specific training was associated with higher self-efficacy but not with objective knowledge, while foundational professional qualification showed a univariate association with objective knowledge that did not persist after multivariable adjustment, suggesting the knowledge gap may reflect broader, currently unmeasured curricular or training-delivery factors rather than any single demographic driver - a hypothesis warranting further investigation rather than a confirmed finding. Glucagon administration and Ramadan-specific glycemic counseling emerged as the two lowest-confidence domains among the self-efficacy items assessed, representing potential targets for future structured retraining. Addressing this knowledge-confidence discordance, rather than simply expanding training volume, may be a relevant consideration for primary diabetes care workforce development in Saudi Arabia, though this study did not directly assess clinical performance or patient-safety outcomes.

## Appendices

Appendix 1:** **study questionnaire (English version)

Source: Original instrument developed by the authors of this study.

Primary Care Nurses’ Knowledge, Confidence, and Satisfaction in Diabetes Related Care: A Cross-Sectional Study

Khobar Health Network, Eastern Cluster, Ministry of Health, Saudi Arabia

Researchers:

Dr. Ayat Ali Al Ali

Family Medicine consultant

Dr. Abdulrahman Fouad Alkhateeb

Family Medicine Consultant

Dr. Sanaa Sadiq Alsaad

Family Medicine Consultant and Diabetologist

Dr. Abdullah Albahussain

Family Medicine Specialist

Ahlam Saeed Almadhi

Nursing Technician

Objectives:

1. Assess the current level of knowledge, confidence and satisfaction among primary care nurses in diabetes related care.

2. Identify determinants affecting the level of knowledge, confidence and satisfaction among primary care nurses in diabetes related care.

Research Questions

1. Are Nurses working at PHC, Khobar Health Network, knowledgeable in diabetes care?

2. Are Nurses working at PHC, Khobar Health Network, confident in diabetes care?

3. Are Nurses working at PHC, Khobar Health Network, satisfied in regard to diabetes care?

4. What are the factors affecting Nurses knowledge, confidence and satisfaction level in relation to diabetes care?

5. Are Nurses with chronic care background (experience), more knowledgeable, confident and satisfied in diabetes care in comparison to those without chronic care experience?

6. Does diabetes related care include patient health education needs a specialized nurse with Diabetes related Background or a diabetic educator?

7. Is team-based care model suitable for dealing with diabetes patients with multiple educational needs on different occasions?

Rationale:

1. Primary care nurses are ideally positioned to provide comprehensive diabetes care, which includes health promotion, prevention, and management of complications. However, their level of knowledge, confidence, and satisfaction in this area can significantly impact the quality of care provided. Therefore, it is a very important research topic for improvement of primary health care services.

2. Understanding the knowledge, confidence, and satisfaction levels of primary care nurses in diabetes management is crucial for ensuring high-quality care in Saudi Arabia.

3. Additionally, study results may help in improvement of knowledge, confidence and satisfaction level of primary care nurses in diabetes related care.

4. This study may help in providing recommendations for enhancing knowledge, confidence, and satisfaction levels of primary care nurses in dealing with diabetes to improve the quality of healthcare services.

5. This study may help to prepare educational and training programs for improving the knowledge, confidence and satisfaction of primary care nurses in diabetes related care.

Inclusion:

· All Nurses who are working in primary health care centers, Khobar Health Network.

Exclusion:

· Nurses who are working in specialty clinics in Khobar Health Network.

-Data collection

Data collection tool:

· self-administered questionnaire

· created by research team

· created in response to organizational nurse assessment tool

· knowledge part includes some questions from the Diabetes Basic Knowledge Test (DBKT). [4]

· translated to Arabic

Data collection will be through a link during face-to-face interview after consent to all nurses working at PHC, Khobar Health Network.

Validity:

Score (1-10) 1=strongly unimportant, Unclear                             10=strongly important, Clear

Please answer the following information by choosing one of the choices:

1. Age

        21-30

        31-40

        41-50

        51-60

        >60

2. Gender 

        Female

        Male

3. Number of years in practice:

        < 2

        2-5

        5-10

        11-15

        15-20

        > 20

4. Job description:

        Worked currently as main chronic disease nurse.

        Worked currently as alternative chronic disease nurse

        Worked previously as main chronic disease nurse.

        Worked previously as alternative chronic disease nurse.

        Didn’t work previously as chronic disease nurse.

5. If you have worked currently or previously as a chronic disease nurse, please determine for how long?

_____________________________________

6. Your degree:

        Nurse specialist (Bachelor degree)

        Nurse technician

        Nurse assistant

        Other degree, please specify: __________

7. Received training in taking care of diabetic patient:

        Yes

        No

8. If received training in dealing with diabetic patient, training was:

        within the last year

        within the last two years

        More than 2 years ago.

9. If received training in dealing with diabetic patient, training was in form of:

        Lectures

        Workshops

        Attachment (onsite training in PHC)

        Others, please specify: __________

10. If received training in dealing with diabetic patient, what was the duration of your training? _____________________________________

11. Do you have diabetes?

        Yes

        No

12. Does anyone in your immediate family members have diabetes?

        Yes

        No                               If yes, who_________

Knowledge

Instruction:

· For each item, select the one best answer to the question.

· The last answer to each question, “I don’t know” should be used if you truly do not know the answer.

· Please answer all the questions.

· An asterisk (*) marks the correct response for each knowledge item.

1. What is the fasting blood glucose level in which diabetes is diagnosed?

        more than or equal 100 mg/dl

        more than or equal 116 mg/dl

        more than or equal 126 mg/dl *

        more than or equal 136 mg/dl

        I do not know

2. What is the blood glucose level threshold in defining hypoglycemia in patients known to have diabetes?

        less than 50 mg/dl

        less than 60 mg/dl

        less than 70 mg/dl*

        less than 80 mg/dl

        I do not know

3. Which statement describes the etiology of Type 1 diabetes?

        It is strongly associated with obesity

        It is predominantly genetic

        It is duo to autoimmune, viral or toxic destruction of the beta cells *

        I do not know

4. Which of these statements about managing Type 1 diabetes is true?

        insulin injections are necessary to maintain life *

        insulin injections are not always necessary if diet and exercise are well controlled.

        oral hypoglycemic agents are sufficient for blood control in most patients

        I do not know.

5. Which statement describes the etiology of Type II diabetes?

        It is predominately non-genetic

        It is frequently associated with obesity and resistance to insulin *

        It is duo to autoimmune, viral or toxic destruction of the beta cells

        I do not know

6. Which of these statements about managing of Type II diabetes is true?

        insulin injections are necessary to maintain life

        a controlled diet and exercise program is the most effective treatment. *

        oral anti-diabetic agents are sufficient for blood control in most patients

        I do not know

7. What effect does insulin have on blood glucose?

        insulin causes blood glucose to increase

        insulin causes blood glucose to decrease *

        insulin has no effect on blood glucose

        I do not know

8. If a person with diabetes is found unresponsive (unconscious) which of these assumptions should guide your initial actions?

        the blood sugar may be very high

        the blood sugar may be very low *

        the blood sugar may be normal

        I do not know

9. Which of the following fasting blood glucose level is considered normal in non-diabetic patients:

           Less than 100 mg/dl *

           less than 126 mg/dl

           less than 150 mg/dl

           I do not know

10. Which of the following affects the accuracy and preparation of test results obtained with most of the blood glucose strips?

        size and placement of the blood sample, timing of the test, expiration of test strips

        size and placement of the blood sample, timing of the test, the patient’s hematocrit level

        size and placement of the blood sample, expiration of test strips, the patient’s hematocrit level *

        I do not know.

11. What would a negative urine glucose test indicate in a diabetic patient with a normal renal threshold?

        Blood glucose level is less than 100 mg/dl

        Blood glucose level is less than 200 mg/dl *

        Blood glucose level is less than 60 mg/dl

        I do not know

12. Which of the following tests can determine the patient’s average blood glucose control over an extended period of time (around 3 months)?

        glycosylated hemoglobin (HbA1C). *

        plasma renin activity (PRA)

        insulin antibodies

        I do not know

13. The target fasting blood glucose level in patient with diabetes is between:

        70-120 mg/dl

        80-130 mg/dl *

        90-140 mg/dl

        100-150 mg/dl

        I do not know

14. The target post-prandial blood glucose level in patient with diabetes is:

        less than 200 mg/dl

        less than 180 mg/dl *

        less than 160 mg/dl

        less than140 mg/dl

        I do not know

15. Which of the following is a long-acting insulin?

        Insulin aspart  (NovoLog)  

        Insulin glargine (Lantus, Toujeo)*

        Insulin lispro (Humalog)

        Regular insulin (Humulin R, Novolin R)

        I do not know

16. Which of the following is considered as a symptom of hypoglycemia (low blood sugar):

        frequent urination

        dry mouth and dry skin

        nervousness *

        I do not know.

17. Which of the following is considered as a symptom of hyperglycemia (high blood sugar):

        frequent urination *

        low grade fever

        cool clammy skin

        I do not know.

18. One of the causes of hypoglycemia (low blood sugar) in someone who is taking insulin or oral hypoglycemic agents is:

        skipping a meal *

        emotional stress

        too little exercise

        I do not know

19. One of the causes of hyperglycemia (high blood sugar) in patients with diabetes is:

        decreased food intake

        infection *

        excessive insulin

        I do not know

20. One of the signs of diabetic ketoacidosis is:

        cold, clammy skin

        acetone (fruity) breath *

        negative urine for glucose

        I do not know

21. Among the following choices, what is the most common cause of diabetic ketoacidosis in Type 1 diabetes?

        excessive exercise

        excessive intake of diet soft drinks over a prolonged period

        failure to take daily insulin dose *

        I do not know

22. Which of the following long-term complications are associated with diabetes?

           eye changes

           renal and cardiovascular changes

           nervous system changes

           all of the above *

           I do not know

23. Which of the following describes the effect of physical and emotional stress on blood glucose level?

           Secretion of stress hormones causes an elevation in blood glucose levels. *

           Secretion of stress hormones causes a decrease in blood glucose levels.

           Secretion of stress hormones has no effect on blood glucose levels.

           I do not know.

24. Proper foot care in patients with diabetes is vital because:

           injecting insulin into the thighs for several years can cause edema in both legs and feet.

           flat feet are commonly associated with diabetes unless preventative measures are routinely used.

           Patients with diabetes often have changes in sensation and poor circulation in their feet. *

           I do not know.

25. A person with diabetes has just received a minor abrasion on the left leg. What should be done to treat the abrasion?

        wash gently with mild soap and water, dry with clean towel and observe carefully for any signs of infection. *

        wash gently with mild soap and water, apply a small amount of iodine and observe carefully for any signs of infection.

        apply a small amount of iodine and call the doctor

        I do not know

26. What effect does exercise have on blood glucose when the patient blood glucose level is more than 300 mg/dl?

        decrease blood glucose

        increase blood glucose *

        has little effect on blood glucose

        I do not know

27. What effect does excessive exercise have on the food needs of a patient with well controlled type 1 diabetes?

        decreases the need for food

        increases the need for food *

        has little effect on the need for food

        I do not know

28. In case of hypoglycemia (low blood sugar), which of the following is the most appropriate INITIAL action to be taken by the patient?

        drink 4oz (120 ml) of regular soda or apple juice *

        drink 4 oz (120 ml) of orange juice with 2 tsp of sugar

        eat 4 crackers with butter or margarine

        I do not know 

29. Which of these is the main objective when developing a meal plan for a person with diabetes?

        a nutritionally balanced, six, small-meal-per-day plan that will prevent delayed stomach emptying time.

        an individualized diet plan that will maintain euglycemia and normal growth and development to include foods from the basic              four food groups, while ensuring that calories are evenly distributed to prevent excess weight. *

        a low-fat, low-fiber diet to prevent excessive weight gain and minimize the risk of cardiovascular disease.

        I do not know

30. Which of the following insulins have an onset time of 0-15 minutes?

        Glargine (Lantus, Toujeo)

        NPH (Humalin N, Novolin N)

        Aspart (NovoLog) *

        Regular (Humalin R, Novolin R)

        I do not know 31. Which of the following sets of figures best illustrated the correct sites for subcutaneous insulin administration?

(Choices are not available)

32. The target glycated hemoglobin (HbA1c) for most diabetic patient is:

        less than 7% *

        7-9 %

        9-11%

        I don’t know

33. Glucagon is the preferred treatment for conscious individuals with hypoglycemia:

        True

        False *

        I do not know

34. Pneumococcal vaccine is not recommended for patient with diabetes:

        True

        False *

        I do not know

35. Most adults with diabetes should engage in 100 min or more of moderate-intensity aerobic activity per week:

        True

        False *

        I do not know

36. Modest, sustained weight loss can delay the progression from prediabetes to type 2 diabetes and is beneficial for the management of type 2 diabetes:

        True *

        False

        I do not know

**Table 8:** Study Questionnaire (English Version) Source: Original instrument developed by the authors of this study.

Confidence - Without additional training how confident do you feel in your ability to optimally perform the following activities: Mark from 1-5 1- not confidence 2- Little Confident 3- Neutral 4- Confident 5- 100% Highly Confident										
Questions	1-Not Confident		2-Little Confident		3-Neutral			4-Confident		5-Highly Confident
The ability to measure blood glucose accurately using Glucometer device.										
The ability to inject insulin accurately.										
The ability to give glucagon accurately.										
The ability to identify symptoms and signs of hypoglycemia.										
The ability to take action in management of hypoglycemia.										
The ability to identify symptoms and signs of hyperglycemia.										
The ability to educate patients with diabetes on the essential elements of foot care.										
The ability to educate patients with diabetes about needed dietary changes.										
The ability to educate patients with diabetes about the role of exercise in diabetes management.										
The ability to educate diabetic patient how to self-monitor blood glucose level.										
The ability to educate diabetic patients about insulin and how to administer it.										
The ability to educate diabetic patient how to identify and manage hyperglycemia at home.										
The ability to educate diabetic patient how to identify and manage hyperglycemia at home.										
The ability to educate diabetic patient how to response to the blood sugar changes in sick days.										
The ability to educate diabetic patient how to response to the blood sugar changes in response to exercise.										
The ability to educate diabetic patient how to store and transfer insulin during travel.										
The ability to educate patients on the importance of frequent blood glucose monitoring and how to response to the blood sugar changes in Ramadan.										
Satisfaction - Mark from 1-5 1- Very dissatisfaction 2-Dissatisfied 3-Neutral 4-Satisfied 5- Very satisfied										
Questions		1- Very dissatisfied		2-Dissatisfied		3-Neutral	4-Satisfied		5- Very Satisfied	
Are you satisfied with your knowledge in dealing with diabetic patients?										
Are you satisfied with your practice in dealing with patients with diabetes?										
Are you satisfied with the training you received to care for diabetic patients?										

Appendix 2: study questionnaire (Arabic version)

Source: Original instrument developed by the authors of this study; Arabic translation of Table 8 prepared by the research team.

Survey Tool  (عربي)

الرجاء الإجابة على الأسئلة باختيار أحد الخيارات:

١- العمر

·         ٢١-٣٠ سنة

·         ٣١-٤٠ سنة

·         ٤١-٥٠ سنة

·         ٥١-٦٠ سنة

·         >٦٠ سنة

٢-الجنس

·         انثى

·         ذكر

٣-الخبرة (عدد سنوات العمل في مجال التمريض)

·         <٢

·         ٢-٥

·         ٦-١٠

·         ١١-١٥

·         15-20

·         > 20

٤-وصف طبيعة العمل

·         أعمل حاليا كممرض/ة أساسية في نشاط الأمراض المزمنة

·         أعمل حاليا كممرض/ ة بديلة في نشاط الأمراض المزمنة

·         سبق لي العمل كممرض/ة أساسية في نشاط الأمراض المزمنة

·         سبق لي العمل كممرض/ة بديلة في نشاط الأمراض المزمنة

·         لم يسبق لي العمل كممرض/ة في نشاط الأمراض مزمنة

٥- إذا كنت تعمل حاليا او سبق لك العمل كممرض/ ة في نشاط الأمراض مزمنة, فماهي المدة الزمنية التي قضيتها في النشاط ؟ ------------

٦- المؤهل:

·         أخصائي تمريض

·         فني تمريض

·         مساعد تمريض

·         مؤهل إضافي , الرجاء تحديده ................

٧- هل تلقيت تدريب في رعاية ومتابعة مرضى السكري

·         نعم

·         لا

٨- إذا سبق وتلقيت تدريب في رعاية مرضى السكري, فمتى تلقيت آخر تدريب ؟

·         خلال السنة الماضية

·         في فترة ما بين سنة الى سنتين

·         قبل أكثر من سنتين

        ٩- إذا سبق وتلقيت تدريب في رعاية مرضى السكري٫ فماهي نوعية التدريب الذي تلقيته؟

·         محاضرات

·         ورش عمل

·         تدريب عملي في المركز

·         أخرى , الرجاء اذكرها ---------

١٠- إذا سبق وتلقيت تدريب في رعاية مرضى السكري , فماهي مدة التدريب الذي تلقيته ؟ ---------

١١- هل أنت مصاب بمرض السكري؟ 

·         نعم                    

·         لا

١٢- هل لديك أحد الأقارب من الدرجة الأولى مصاب بمرض السكري

·         نعم

·         لا

·         إذا كانت الإجابة بنعم ٫ فمن المصاب .......................

 (مستوى المعرفة)Knowledge

تعليمات

·         بالنسبة لكل سؤال، حدد أفضل إجابة على السؤال.

·         ينبغي استخدام الإجابة الأخيرة على كل سؤال "لا أعلم" إذا كنت لا تعرف الإجابة حقًا.

·         الرجاء الإجابة على جميع الأسئلة

١-ما هو مستوى تحليل السكر صائم اللازم لتشخيص مرض السكري

·         أكثر من أو يساوي ١٠٠ ملجم/دل

·         أكثر من أو يساوي ١١٦ ملجم/دل

·         أكثر من أو يساوي ١٢٦ ملجم/دل *

·         أكثر من أو يساوي ١٣٦ ملجم/دل

·         لا أعلم

٢-ما هو مستوى تحليل السكر اللازم لتعريف هبوط السكر لدى المرضى المصابين بالسكري

·         أقل من ٥٠ ملجم/دل

·         أقل من ٦٠ ملجم/دل

·         أقل من ٧٠ ملجم/دل *

·         أقل من ٨٠ ملجم/دل

·         لا أعلم

٣- ما هي العبارة التي تصف مسببات مرض السكري ( النوع الأول ) ؟

·         السبب له علاقة قوية بالسمنة

·         السبب في الغالب جيني

·         السبب هو خسارة خلايا بيتا نتيجة للمناعة الذاتية أوالتعرض لبعض أنواع الفيروسات أو السموم*

·         لا أعلم

٤-أي من هذه العبارات حول علاج مرض السكري من النوع الأول صحيحة؟

·         حقن الأنسولين ضرورية للحفاظ على الحياة *

·         حقن الأنسولين غير ضرورية دائمًا إذا تم التحكم جيدًا في النظام الغذائي وممارسة الرياضة

·         أدوية خفض السكر عن طريق الفم كافية للتحكم في السكر لدى معظم المرضى

·         لا أعلم

٥-ما هي العبارة التي تصف مسببات مرض السكري (النوع الثاني) ؟

·         الأسباب في الغالب غير وراثية

·         الأسباب في الغالب مرتبطة بالسمنة ومقاومة الأنسولين *

·         خسارة خلايا بيتا نتيجة للمناعة الذاتية أوالتعرض لبعض أنواع الفيروسات أو السموم

·         لا أعلم

٦-أي من هذه العبارات حول علاج  مرض السكري من النوع الثاني صحيحة؟

·         حقن الأنسولين ضرورية للحفاظ على الحياة

·         اتباع نظام غذائي وممارسة التمارين الرياضية هو العلاج الأكثر فعالية *

·         أدوية خفض السكر عن طريق الفم كافية للتحكم في السكر لدى معظم المرضى

·         لا أعلم

٧-ما هو تأثير الأنسولين على نسبة الجلوكوز في الدم؟

·         الأنسولين يسبب زيادة نسبة السكر في الدم

·         الأنسولين يسبب انخفاض نسبة السكر في الدم *

·         ليس للأنسولين أي تأثير على نسبة الجلوكوز في الدم

·         لا أعلم

٨-إذا تبين أن الشخص المصاب بمرض السكري لا يستجيب ( فاقد للوعي )، أي من هذه الافتراضات يجب أن توجه رد فعلك الإسعافي الأول؟

·         قد يكون مستوى السكر في الدم مرتفعًا جدًا

·         قد يكون مستوى السكر في الدم منخفضًا جدًا *

·         قد يكون مستوى السكر في الدم طبيعيا

·         لا أعلم

٩- أفضل وصف لمستوى السكر في الدم أثناء الصيام لدى المرضى غير المصابين بالسكري هو:

·         أقل من 100 ملجم/ديسيلتر*

·         أقل من 126 ملجم/ديسيلتر

·         أقل من 60 ملجم/ديسيلتر

·         لا أعلم

١٠- أي مما يلي يؤثر على دقة وإعداد نتائج الاختبار التي يتم الحصول عليها باستخدام معظم شرائح الجلوكوز في الدم ؟

·          حجم عينة الدم وموضعها , توقيت الاختبار , انتهاء صلاحية شرائط الاختبار

·         حجم عينة الدم وموضعها , توقيت الاختبار , مستوى الهيماتوكريت لدى المريض

·         حجم عينة الدم وموضعها , انتهاء صلاحية شرائط الاختبار, مستوى الهيماتوكريت لدى المريض

·         لا أعلم

١١- ما الذي تدل عليه النتيجة السلبية لاختبارالجلوكوز في البول لدى مريض السكري الذي لديه وظائف كلوية طبيعية ؟

·         مستوى الجلوكوز في الدم أقل من ١٠٠ ملجم/ديسيلتر

·         مستوى الجلوكوز في الدم أقل من ٢٠٠ ملجم/ديسيلتر *

·         مستوى الجلوكوز في الدم أقل من ٦٠ ملجم/ديسيلتر

·          لا أعلم

١٢- أي من الاختبارات التالية يمكنه تحديد مستوى في الدم  لدى المريض على مدى فترة زمنية ممتدة (حوالي ثلاثة أشهر )؟

·         الهيموجلوبين الغليكوزيلاتي (HbA1C) *

·         نشاط الرينين في البلازما  (PRA)

·         الأجسام المضادة للأنسولين

·         لا أعلم

١٣- مستوى السكر الصائم المستهدف لدى مريض السكري:

·         ٧٠-١٢٠ ملجم/ديسيلتر

·         ٨٠-١٣٠ ملجم/ديسيلتر *

·          ٩٠-١٤٠ ملجم/ديسيلتر

·         ١٠٠-١٥٠ملجم/ديسيلتر

·         لا أعلم

١٤- مستوى السكرالمستهدف بعد الأكل بساعتين لدى مريض السكري هو:

·         أقل من ٢٠٠ ملجم/ديسيلتر

·         أقل من ١٨٠ ملجم/ديسيلتر *

·         أقل من ١٦٠ ملجم/ديسيلتر

·         أقل من ١٤٠ ملجم/ديسيلتر

١٥- أي من أنواع الانسولين التالية يعتبر انسولين طويل المفعول ؟

·         Insulin aspart   (NovoLog)  

·         Insulin glargine (Lantus , Toujeo)*

·         Insulin lispro (Humalog)

·         Regular insulin (Humulin R, Novolin R)

·         I do not know

١٦- من أعراض انخفاض السكر في الدم :

·         كثرة التبول

·         جفاف الفم وجفاف الجلد

·         العصبية *

·         لا أعلم

١٧- من أعراض ارتفاع السكر في الدم:

·         كثرة التبول *

·         حمى منخفضة الدرجة

·         بشرة باردة ورطبة

·         لا أعلم.

١٨- ما هو أحد أسباب هبوط السكر في الدم (انخفاض نسبة السكر في الدم) لدى الشخص الذي يأخذ الأنسولين أو أدوية السكر عن طريق الفم؟

·         تخطي وجبة *

·         التوتر

·          قلة ممارسة الرياضة

·         لا أعلم

١٩- ما هو أحد أسباب ارتفاع السكر في الدم لدى مريض السكري؟

·         قلة تناول الطعام

·         إصابة بعدوى أو التهاب  *

·         الإفراط في جرعة الانسولين

·         لا أعلم

٢٠- من العلامات المصاحبة للحموضة الكيتونية السكرية :

·         الجلد البارد والرطب

·         نفس الأسيتون (الفواكه)*

·         تحليل البول سلبي للجلوكوز

·          لا أعلم

٢١- أي مما يلي يعد السبب الرئيسي للحموضة الكيتونية السكرية لدى مرضى السكري من النوع الأول ؟

·         الإفراط في الرياضة

·         الإفراط في تناول المشروبات الغازية الخاصة بالحمية على مدى فترات طويلة

·         عدم أخذ حقن الانسولين *

·         لا أعلم

٢٢- أي من المضاعفات طويلة المدى التالية ترتبط بمرض السكري؟

·         مضاعفات في العين

·         مضاعفات في الكلى والقلب والأوعية الدموية

·         مضاعفات  في الجهاز العصبي

·         كل ما ورد أعلاه *

·          لا أعلم

٢٣- أي مما يلي يصف أثرالضغوط الجسدية والعاطفية على مستوى السكر في الدم لدى مريض السكري ؟

·         إفراز هرمونات التوتر التي تسبب ارتفاع مستوى السكر في الدم.*

·         إفراز هرمونات التوتر التي تسبب انخفاض مستوى السكر في الدم.

·         إفراز هرمونات التوتر التي ليس لها أي تأثير على مستويات السكر في الدم

·         لا أعلم

٢٤- العناية الجيدة بالقدم لدى مريض السكر مهمة للغاية بسبب ؟

·         حقن الأنسولين في الفخذين لعدة سنوات ممكن أن يسبب وذمة (تورم أو انتفاخ) في الساقين والقدمين.

·          ترتبط الأقدام المسطحة عادةً بمرض السكري ما لم يتم استخدام التدابير الوقائية بشكل روتيني.

·          غالبًا ما يعاني الأشخاص المصابون بداء السكري من تغيرات في الإحساس وضعف الدورة الدموية في أقدامهم.*

·          لا أعلم.

٢٥- أصيب شخص مصاب بالسكري بخدش بسيط في ساقه اليسرى. ما الذي يجب فعله لعلاج الجرح؟

·         غسله بلطف بالماء والصابون ، ثم تجفيفه بمنشفة نظيفة ومراقبته بعناية بحثا عن أي علامات للعدوى. *

·         غسله بلطف بالماء والصابون ، ثم وضع كمية صغيرة من اليود ومراقبته بعناية بحثا عن أي علامات للعدوى

·         وضع كمية صغيرة من اليود ثم زيارة الطبيب

·         لا أعلم

٢٦- ما هو تأثير التمارين الرياضية عند مرضى السكري على نسبة الجلوكوز في الدم عندما يكون مستوى الجلوكوز في الدم أكثر من ٣٠٠ ملغم / ديسيلتر؟

·         انخفاض نسبة الجلوكوز في الدم

·         زيادة نسبة الجلوكوز في الدم *

·         ليس له تأثير يذكر على نسبة الجلوكوز في الدم

·         لا أعلم

٢٧- ما هو تأثير الإفراط في ممارسة التمارين الرياضية على الاحتياجات الغذائية للشخص المصاب بداء السكري من النوع الأول المتحكم فيه بشكل جيد؟

·         تقليل الحاجة إلى الطعام

·         يزيد الحاجة إلى الطعام *

·         ليس له تأثير يذكر على الحاجة إلى الطعام

·         لا أعلم

٢٨- ما هو الإجراء الأولي الأكثر ملاءمة الذي يجب اتخاذه بالنسبة لشخص مصاب بالسكري ويعاني من انخفاض نسبة السكر في الدم؟

·         شرب١٢٠ مل من الصودا العادية أو عصير التفاح *

·         شرب ١٢٠ مل من عصير البرتقال مع ملعقتين صغيرتين من السكر

·         تناول ٤ قطع من البسكويت مع الزبدة أو السمن

·         لا أعلم

٢٩- أي مما يلي هو الهدف الرئيسي عند وضع خطة وجبات لشخص مصاب بالسكري؟

·         خطة غذائية متوازنة مكونة من ستة وجبات صغيرة يوميًا تمنع تأخير إفراغ المعدة.

·          خطة نظام غذائي فردية تحافظ على سكر الدم والنمو الطبيعي والتطور لتشمل الأطعمة من المجموعات الغذائية الأربع الأساسية، مع ضمان توزيع السعرات الحرارية بالتساوي لمنع الوزن الزائد. *

·         اتباع نظام غذائي قليل الدهون ومنخفض الألياف لمنع زيادة الوزن الزائد وتقليل خطر الإصابة بأمراض القلب والأوعية الدموية.

·         لا أعلم

٣٠- أي من أنواع الأنسولين التالية يبدأ مفعولها خلال ١٥ دقيقة :

·         Glargine (Lantus , Toujeo)

·         NPH (Humalin N, Novolin N)

·         Aspart (NovoLog) *

·         Regular (Humalin R, Novolin R)

·         لا أعلم

٣١- أي من مجموعات الأشكال التالية توضح المواقع الصحيحة لإعطاء الأنسولين تحت الجلد؟

٣٢- مستوى السكر التراكمي المستهدف (HbA1c) لمعظم مرضى السكري هو:

·         أقل من٧٪ *

·         من٧٪-٩٪

·         ٩ -١١٪

·         لا أعلم

٣٣- الجلوكاجون هو العلاج المفضل لشخص واعي مصاب بانخفاض السكر في الدم:

·         صحيح

·         خطأ *

٣٤- لا يوصى بتطعيم المكورات الرئوية (pneumococcal vaccine) لمريض السكري:

·         صحيح

·          خطأ *

·          لا أعلم

٣٥- ينصح معظم البالغين المصابين بالسكري بممارسة ١٠٠ دقيقة أو أكثر من الأنشطة الرياضية الهوائية متوسطة الشدة أسبوعيًا:

·         صحيح

·          خطأ *

·          لا أعلم

٣٦- يمكن لفقدان الوزن بشكل متوسط ومستمر أن يؤخر تطورمرحلة ما قبل السكري  (prediabetes)إلى مرحلة مرض السكري من النوع الثاني وهو مفيد لإدارة مرض السكري من النوع الثاني:

·         صحيح *

·         خطأ

·         لا أعلم

 (مستوى الثقة)Confidence

بدون الحصول على تدريب إضافي، ما مدى شعورك بالثقة في قدرتك على أداء الأنشطة التالية على النحو الأمثل:

العلامة من ١ إلى ١٠

١ = عدم الثقة.

٥ = ١٠٠٪ واثق ( ثقة تامة )

**Table 9:** Study Questionnaire (Arabic Version) Source: Original instrument developed by the authors of this study.

(مستوى الثقة)Confidence بدون الحصول على تدريب إضافي، ما مدى شعورك بالثقة في قدرتك على أداء الأنشطة التالية على النحو الأمثل: العلامة من ١ إلى ١٠ ١ = عدم الثقة. ٥ = ١٠٠٪ واثق ( ثقة تامة )										
٥واثق تماما	٤واثق			٣محايد	٢واثق قليلا		١ غير واثق أبدا			
									١-التمكن من قياس نسبة الجلوكوز في الدم بدقة باستخدام جهاز Glucometer	
									٢-التمكن من حقن الأنسولين بشكل صحيح	
									٣-التمكن من حقن الجلوكاجون بشكل صحيح	
									٤-التمكن من تحديد أعراض وعلامات هبوط (إنخفاض) السكر في الدم لمريض السكري	
									٥-التمكن من اتخاذ الإجراءات اللازمة لعلاج هبوط (إنخفاض) السكر في الدم	
									٦-التمكن من تحديد أعراض وعلامات ارتفاع السكر في الدم لمريض السكري	
									٧-التمكن من تثقيف مريض السكري حول العناصر الاساسية في العناية الجيدة بالقدم	
									٨-التمكن من تثقيف مرضى السكري حول التغيرات اللازمة في نمط الغذاء ٩-التمكن من تثقيف مرضى السكري حول أهمية الرياضة في علاج مرض السكري	
									١٠-التمكن من تثقيف مريض السكري عن كيفية المراقبة الذاتية لمستوى السكر في الدم	
									١١-التمكن من تثقيف مرضى السكري عن الأنسولين وكيفية استخدامه	
									١٢-التمكن من تثقيف مريض السكري عن كيفية التعرف على هبوط السكر و علاجه في المنزل	
									١٣-التمكن من تثقيف مريض السكري كيفية التعرف على ارتفاع السكر و علاجه في المنزل	
									١٤-التمكن من تثقيف مريض السكري عن كيفية الاستجابة لتغيرات السكر في الدم خلال أيام المرض	
									١٥-التمكن من تثقيف مريض السكري عن كيفية الاستجابة لتغيرات السكر في الدم أثناء ممارسة الرياضة	
									١٦-التمكن من توعية مريض السكري عن كيفية تخزين ونقل الأنسولين أثناء السفر	
									١٧-التمكن من توعية مرضى السكري بأهمية المتابعة الدورية لمستوى السكرو كيفية الاستجابة لتغيرات مستوى السكر أثناء شهر رمضان المبارك	
(مستوى الرضا)Satisfaction اختر من ١ إلى ١٠ ١= مستاء بشدة ٥= راضٍ بشدة ١٠٠٪										
٥راضٍ بشدة		٤راضٍ	محايد ٣			٢مستاء		١مستاء بشدة		
										١-هل أنت راضٍ عن مستوى معرفتك الخاصة بالتعامل مع مرضى السكري؟
										٢-هل أنت راضٍ عن مستوى ممارستك الخاصة بالتعامل مع مرضى السكري؟
										٣-هل أنت راضٍ عن التدريب الذي تلقيته الخاص برعاية مرضى السكري؟
